# Nanostructured P3HT as a Promising Sensing Element for Real-Time, Dynamic Detection of Gaseous Acetone

**DOI:** 10.3390/s19061296

**Published:** 2019-03-14

**Authors:** Cristina Bertoni, Pasquale Naclerio, Emanuele Viviani, Simone Dal Zilio, Sergio Carrato, Alessandro Fraleoni-Morgera

**Affiliations:** 1 Global Connectivity & Technology—Robotics and Artificial Intelligence, Corso Lino Zanussi 24, 33080 Porcia (PN), Italy; cristina.bertoni@electrolux.com; 2 Department of Engineering and Architecture, University of Trieste, Via Valerio 10, 34127 Trieste, Italy; naclerio.pasquale@gmail.com (P.N.); carrato@units.it (S.C.); 3 Artificial Perception Laboratory, Department of Engineering and Architecture, University of Trieste, Via Valerio 10, 34127 Trieste, Italy; EMANUELE.VIVIANI@phd.units.it; 4 CNR-Istituto Officina dei Materiali, Strada Statale 14 km 163.5, 34149 Basovizza, Trieste (TS), Italy; dalzilio@iom.cnr.it; 5 Flextronics Laboratory, Department of Engineering and Architecture, University of Trieste, Via Valerio 10, 34127 Trieste, Italy

**Keywords:** gas sensors, P3HT, semiconducting polymer, nanostructured sensors, acetone detection, breath analysis

## Abstract

The dynamic response of gas sensors based on poly(3-hexylthiophene) (P3HT) nanofibers (NFs) to gaseous acetone was assessed using a setup based on flow-injection analysis, aimed at emulating actual breath exhalation. The setup was validated by using a commercially available sensor. The P3HT NFs sensors tested in dynamic flow conditions showed satisfactory reproducibility down to about 3.5 ppm acetone concentration, a linear response over a clinically relevant concentration range (3.5-35 ppm), excellent baseline recovery and reversibility upon repeated exposures to the analyte, short pulse rise and fall times (less than 1 s and about 2 s, respectively) and low power consumption (few nW), with no relevant response to water. Comparable responses’ decay times under either nitrogen or dry air suggest that the mechanisms at work is mainly attributable to specific analyte-semiconducting polymer interactions. These results open the way to the use of P3HT NFs-based sensing elements for the realization of portable, real-time electronic noses for on-the-fly exhaled breath analysis.

## 1. Introduction

The sensing elements of gas sensors based on polymers are often constituted by the so-called intrinsically conducting polymers (ICPs), as either doped conjugated polymers presenting an inherent conductivity or conducting polymer composites with carbonaceous materials [1,2]. These materials are deposited in the form of granular films onto electrodes. When the gaseous analyte adsorbs and penetrates within the grains, it causes a change in the ICP/composite workfunction, which in turn leads to a change in the current detected between the electrodes [3,4,5,6]. Due to this detection mechanism, these devices are termed "chemiresistors". The response times of these systems, due to the penetration of the analyte in the sensing layer, is rather long, in the range of several minutes, which can be decreased to less than one minute by nanostructuring the sensing layer [7,8]. However, even in these cases the full reversibility of the sensor response is not achieved, as at least part of the analyte is retained within the granular polymer/composite layer [9,10].

Semiconducting polymers (SPs) are less explored for gas sensing than ICPs. Usually, SPs are included in field-effect transistors (FETs), as the channel placed between source and drain [11,12], while their use as simple resistors (i.e., SP-based chemiresistors) between two electrodes received scarce attention until now [13,14,15]. Also SPs-based sensors usually suffer from slow response (average response times ranging around tens of seconds in the best cases [11,12,15,16,17]) and little reversibility, with overall performances similar to those of their ICPs-based counterparts. To the knowledge of the authors, the only works reporting gas-sensing performances from SP-based chemiresistors with response time below one minute and satisfactory reproducibility are based on self-assembled nanofibers of P3HT (head-to-tail, regioregular poly(3-hexylthiophene), a well-known semi-conducting polymer), that demonstrated recovery times below one second and nearly perfect reversibility upon exposure to gaseous H_2_O, NH_3_, acetone [18,19], even though the measurements were carried out only at a qualitative level. This performance in terms of rapidity of response was much higher than that of previously reported thin films-based sensors based on the same polymer [13,14,15,16].

Gaseous cetone, as analyte, holds a considerable diagnostic importance due to its presence in human breath, as a marker for diseases like diabetes. As a consequence, a number of devices have been developed for its detection in breath [20,21,22]. Since many different compounds are present in breath (up to more than 1000 [23]), most often this type of analysis is carried out with expensive equipment, like chromatographic systems coupled to mass spectrometry, or to spectrophotometric instruments [23,24]. However, these instruments are time-consuming, operator-dependent and unhandy, and cannot be used for real-time, continuous measurements. In this view, solid-state, simple, low cost and portable devices for real-time and user-friendly breath analysis would be desirable to enable frequent testing for effective diabetes management and treatment. Indeed, solid-state devices able to selectively detect acetone concentration in the sub-ppm range in air have been reported [25,26,27,28], but these sensors are based on metal oxides, that require power consumptions in the range of micro-milliWatts (needed to operate at 300–400 °C for achieving reasonable sensitivity, and to effectively desorb the analyte), and have response times in the order of tens of seconds. Nonetheless, this type of sensors is widely used in electronic noses (i.e., matrices of sensors in which every single sensing element has a higher selectivity for a different component of the analyzed mixture) [29,30], which can discern several different components in a gaseous analyte mixture.

Another feature required for real-time detection of acetone in exhaled breath is the operation in a dynamic gaseous flow, rather than in a quasi-equilibrium environment. In fact, the exhaled breath is essentially a pulsed flow, with each pulse normally lasting for a few seconds. Flow injection analysis (FIA), usually applied mainly to liquid analytes, rather than to gaseous ones, operates in a dynamic fashion, exposing the sensor to the analyte for a limited time, hence not in an equilibrium condition [31]. In some cases, FIA has been applied also to gaseous compounds like CO, ethanol, hydrocarbons, ethylene [32,33,34], and recently organic-based nanostructures have been used for the detection of humidity in a FIA-based setup, showing outstanding performances in terms of response speed (in range of a few tens of milliseconds) and repeatability [35,36,37].

In this work, we assess the performance of P3HT nanostructures-based chemiresistive sensing elements towards gaseous acetone, using a by purpose-developed measuring setup that simulates the breath dynamic behaviour (FIA approach). The sensors have been fabricated using a low-cost and fast procedure named ASB-SANS (Auxiliary Solvent-Based Self-Assembled NanoStructuring) [38,39,40], and operated with a total power of a few nW. The tested acetone concentrations ranged between about 3.5 and 35 ppm (with the lower part of this range being clinically relevant for breath analysis [41]), verifying, in a continuous carrier gas flow, the reliability, reversibility, sensitivity and linearity of the sensing element with respect to the injected amounts of gas. The normalized baseline recovery times have also been assessed. The sensing element evidenced a weak, but detectable, response to water, which however was not seen as meaningfully influencing the response to acetone. The same device has been operated under either nitrogen or air as gas carriers, delivering in both cases comparable performances in the acetone detection, and an analysis of the sensor’s signal decay times operated under both the two different carrier gases has been conducted. On these grounds, some considerations about the possible mechanism of the acetone detection operated by the sensing element are made, and an outlook on the applicability of the P3HT NF-based sensor in the frame of electronic noses for breath detection is given.

## 2. Materials and Methods

### 2.1. Testing System Setup

**Figure 1:**
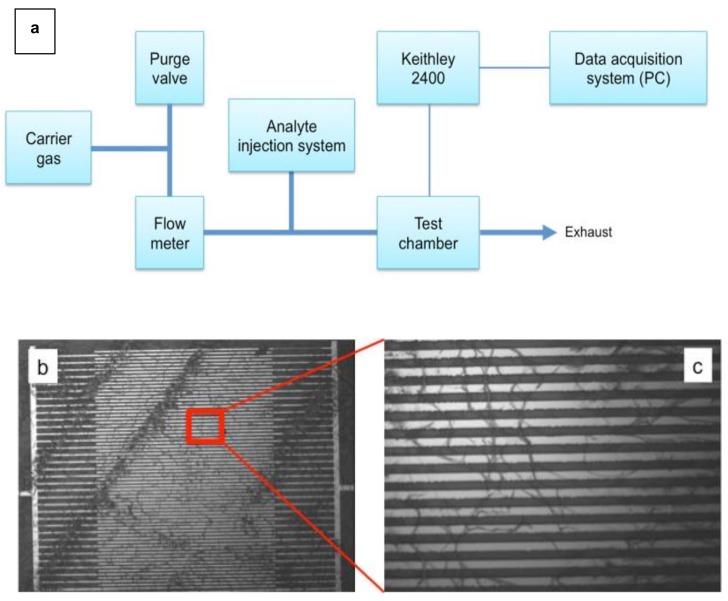
(**a**): Sketch of the gas sensing system used to test sensor devices. (**b**,**c**): micrographs at different magnfications of the tested P3HT NFs sensing element. The sizes of the micrographs are 580 × 435 μm (**b**) and 116 × 86.6 μm (**c**).

The testing setup consists in gas cylinders containing the carrier gases connected to a cylindrical, stainless steel test chamber of about 190 mL of volume. Dry nitrogen or dry, compressed air, both with humidity percentage < 0.1%, have been used as carrier gases. The cross-section ratio between the gas inlet tube and the testing chamber is 1:10, with the testing chamber having a diameter of about 50 mm. Inside the chamber the actual sensor is mounted onto a rigid support housing also the sensor electrical wires (naked copper, to avoid any unwanted effect of analyte absorption from the plastic insulation of cables) and the feed-through connectors (BNC) to the external data acquisition system. A flow meter capable of a 2 to 20 L/min range was added between the cylinders and the test chamber to control the flow of the carrier gas (Figure 1a).

This flow was kept constant along experiments to allow sensors characterization towards injections of small analyte volumes. A flow rate of 10 L/min of N_2_ was used for the setup validation carried out by using the Figaro sensor; such a high flow rate was necessary since using nitrogen as the carrier gas resulted in very slow Figaro response characteristics (see Section 3.1), and flow rates lower than 10 L/min were seen to be unpractical. A flow rate of 2 L/min was instead kept for both N_2_ and air carrier gases for P3HT NFs sensors tests, because this is a typical value for a calm breath exhalation velocity [42]. A purge glycerin valve was placed between the carrier gas source and the test chamber to avoid pressure surges. The gas analyte was delivered via a glass syringe using a dedicated injection connector placed on the gas line. Test parameters and data were set/acquired by a Keithley 2400 SourceMeter connected (Tektronix Inc., Beaverton, OR, USA) to a PC.

### 2.2. Analyte Preparation and Injection Procedure

Sealed vessels containing a small amount of liquid analyte (acetone - electronic grade solvent, or de-ionized water) were left in thermal equilibrium (at least one hour) with the surrounding environment, to allow the formation of saturated vapors of the gas within the vessels themselves. These saturated vapors were used to condition a glass syringe. The same syringe was then used to withdraw fixed volumes of namely 1, 3, 5 and 10 mL of the selected gaseous analyte saturated vapors and to inject them into the system. Injections were carried out manually, at an approximate rate of 1.5–1.8 mL/s (the highest amounts of analyte were injected slightly faster than the lower amounts). The time elapsed between the end of the analyte injection and the onset of the sensor response was measured with a manual chronometer.

The amount of gas injected in the system was determined using the gas law (acetone saturated vapor pressure of 28.6 kPa and water saturated vapor pressure of 3.18 kPa, both at 25 °C). The number of moles per injected mL was then divided by the volume of the test chamber to derive approximate average gas concentrations within the chamber (vide infra for a more detailed discussion on the assumptions underlying this estimate). By applying this method, 1, 3, 5 and 10 mL acetone injections resulted in concentrations corresponding to 3.5, 10.5, 17.6 and 35 ppm, respectively, and 5 and 10 mL injectons of water resulted in concentration of 0.61 and 1.21 ppm, respectively. An overview of the experimental conditions in which the analytes were injected in the system is given in Table 1.

### 2.3. Test Chamber Validation Procedure

The test chamber was validated using a commercial Figaro sensor (TGS826, Figaro, Arlington Heights, IL, USA), operated in dry nitrogen as the carrier gas, using a 10 L/min flow rate. Laboratory air volumes of 1, 5 and 10 mL were injected in the system. The response of the Figaro sensor to these aliquots, expressed in terms of resistance change, was recorded and analyzed in the frame of the dynamic gas flow approach (vide infra).

### 2.4. P3HT Nanofibers-Based Sensors Fabrication and Operation

Interdigitated Au electrodes onto SiO_2_/Si substrates, consisting of identical fingers having 10 μm width and gap, were fabricated at the Trieste CNR-IOM premises using standard lithographic techniques. P3HT nanofibers were grown directly on these substrates according to the procedure described in previous works [18,19,39]. In more detail, 2 μL of a solution composed by 1 mg of P3HT (99% HT, Rieke Metals, Lincoln, NE, USA), 1 mL of CHCl_3_ (spectroscopic grade, Sigma Aldrich, St. Louis, MO, USA) and 0.9 g of *para*-dichlorobenzene (98%, Sigma Aldrich, Saint Louis, MO, USA, spectroscopic grade) were drop cast onto the interdigitated gold electrodes. The deposited solution has been allowed to evaporate, until P3HT nanofibers developed onto the sensor surface (approx. 20 min). The so-fabricated sensors have been used with no further treatment, and operated at 0.5 V and 2 L/min of carrier gas (either dry nitrogen or dry compressed air) flow rate.

## 3. Results

### 3.1. Validation of the Sensor Response

Previous studies showed that P3HT nanofibers (NF)-based CRs exposed to acetone have very fast response times and nearly perfect baseline recovery [18,19]. These results were obtained in a qualitative way, i.e., by blowing acetone vapors onto the sensing layer, in an open environment, realizing in this way a dynamic interaction between the analyte and the sensing surface. Though useful for a first assessment of the device, this procedure called for a more strict control over both the analyte delivery procedure and the delimitation of the overall volume in which the sensing element operates, still conserving the dynamic characteristic of the analyte approach to the sensor’s surface. In fact, as mentioned above, acetone is dynamically exhaled in breath, in its gaseous form. Therefore, a quantitative determination of gaseous acetone aiming to find application in real-time breath analysis must be carried out in a dynamic environment, not in a steady gas flow. In this frame, we decided to characterize the sensitivity of P3HT NFs-based sensors to gaseous acetone using a flow injection analysis approach, which mimics a dynamic environment [31]. A dedicated setup has been hence prepared for this purpose, as described in Section 2.1.

**Figure 2:**
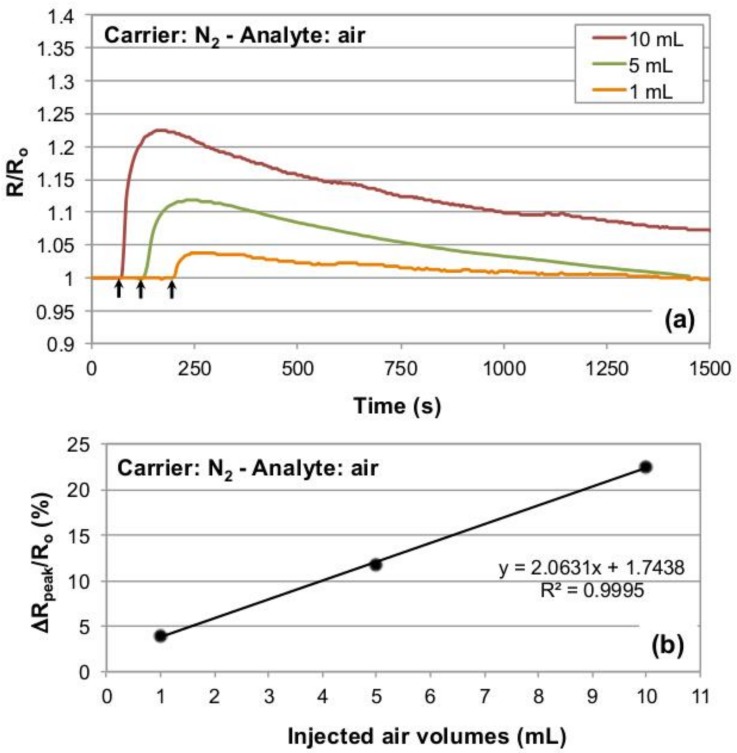
(**a**) Normalized resistance traces obtained from a Figaro commercial sensor operated in nitrogen and exposed to different amounts of laboratory air at 25 °C. As it can be noticed, increasing injected volumes result in progressively higher peak heights. The black arrows mark the end of the analyte injection procedure (approximately three-four seconds prior to observe the sensor response onset. This relatively long time is attributed to the steel mesh protecting the Figaro sensing surface, see the Discussion part for details). (**b**) Percentage resistance change with respect to baseline for the injected gas analyte volumes used to validate the testing chamber.

To validate the response of our experimental setup for dynamic gaseous flow injection analysis, a commercial semiconductor gas sensor (Figaro TGS826) was used as in-line, flow-through detection device [31]. The sensor was operated under a constant nitrogen flow as the carrier gas, injecting 1, 5 and 10 mL of laboratory air as the gas analyte (see Section 2 for details). At a flow rate of the carrier gas of 10 L/min the response behavior of the Figaro was found to be rather slow (Figure 2); however, for lower flow rates of the nitrogen carrier gas the response times were excessively slow, incompatible with reasonable experimental times.

Results of the test chamber validation are reported in Figure 2a, in which the Figaro resistance data recorded for the different injected air aliquots have been normalized to the baseline resistance (R_0_) of the sensor to compare peak maxima. The latter are proportional to the three different injected air volumes. Indeed, the sensor resistance increases in the presence of air, and the recorded peak maxima show a linear relationship with the injected air volumes (R^2^ = 0.9995, Figure 2b), thus assessing the suitability of our test chamber, in the mentioned conditions, for dynamic gaseous analyte detection.

### 3.2. Preliminary Acetone Sensing Tests

Preliminary tests carried out on P3HT NF sensors able to detect gaseous acetone evidenced how by applying a remarkably low bias of 0.5 V, devices were characterized by currents in the order of tens of nA, and by a noise (derived from the standard deviation of the baseline signal) about four orders of magnitude lower [18,19]. In order to make a more quantitative assessment of these parameters in conditions as similar as possible to those found in breath exhalation, a P3HT NF-based sensor was placed in the flow-injection analysis test chamber. A carrier flow rate of 2 L/min, in line with typical velocities of exhaled breath (which are in the range between about 1 and 3 L/min [42]), was used. The P3HT NF-based device was hence exposed to repeated injections of small volumes (1 mL) of acetone. To derive an approximate value of the actual acetone concentration detected by the sensor, we considered that at 2 L/min the carrier gas will take at least 5.8 s to flush the testing chamber. As gas pulses recorded for 1 mL injections are shorter (around 2.5 s) and the regime of gas flow within the cylindrical chamber at 2 L/min is turbulent (a Reynolds number of 54000 for standard air at 25 °C can be derived for the cylindrical testing chamber in the above mentioned conditions; this value has to be compared with that of 2000 accepted for turbulent flow onset), we assumed that the whole volume of injected gas undergoes complete and effective mixing inside the chamber due to turbulence effects, even though detailed investigations over the analyte diffusion profile within the chamber were not carried out. From these considerations, we infer that the maximum concentration of the analyte achievable in the chamber can be approximated by the n° of moles of the injected analyte divided by the chamber volume. Under these considerations, 1 mL of acetone saturated vapors corresponds to about a 3.5 ppm concentration. Therefore, we will consider this value as the actual one, even though we are aware that it could be lower, due to the constant dilution action exerted by the carrier gas flow.

**Figure 3:**
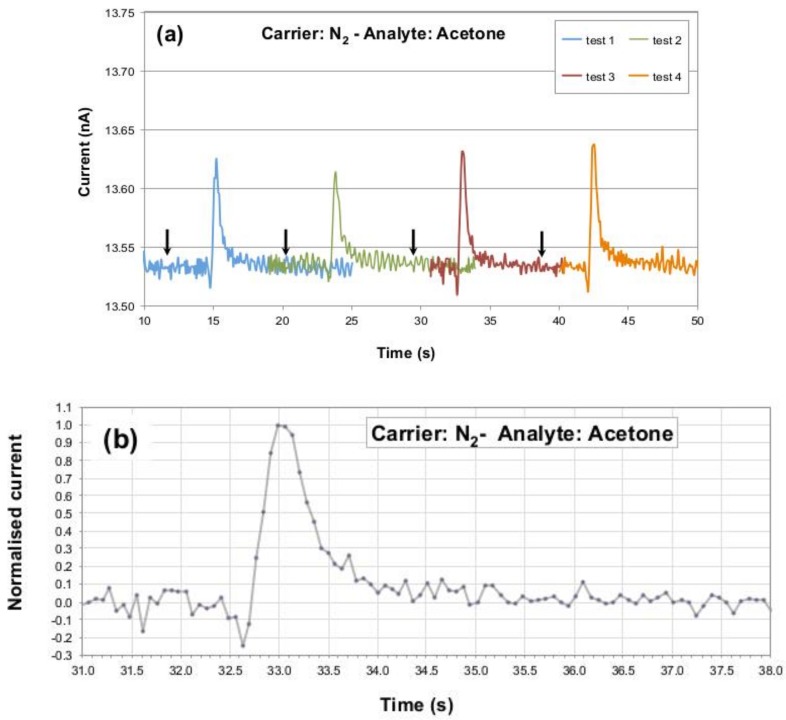
(**a**) Repeated tests for P3HT nanofibers sensors against 3.5 ppm of acetone in nitrogen as carrier gas at 25 °C. (**b**) Normalised current data collected from a P3HT nanofibers-based sensor exposed to 3.5 ppm of acetone in nitrogen as gas carrier at 25 °C. In panel (a) the black arrows mark the end of the analyte injection procedure (approximately 3 s prior to observe the sensor response onset). In panel (b) the injection occurred at around second 30 (not shown, to evidence the perfect baseline recovery occurring in a few seconds after the signal peak).

Figure 3a collects sensor’s current peaks detected during these repeated exposures and shows how the response of the P3HT NF device is reproducible in terms of signal’s maximum, background noise, pulse rise and fall times, and baseline recovery. The small variability observed in the signals maxima is attributed to the manual procedure used for the analyte injection. Figure 3b reports a normalized P3HT NF response (in terms of recorded current) to one injection of the 3.5 ppm *aliquota* of acetone, highlighting (i) the sensor dynamics towards the analyte gas passage with a peak-to-noise ratio of around 38 dB and (ii) pulse rise and fall times (defined as times needed to go from 10 up to 90% and from 90 down to 10% of the peak, respectively) of less than 1 and around 2 s, respectively. The time lapse between the analyte injection start and the sensor response onset was found to be around three seconds.

### 3.3. Sensing Element Calibration Tests

**Figure 4:**
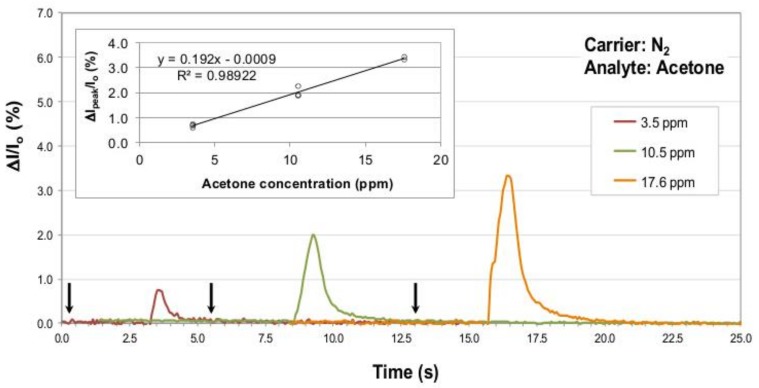
Current percentage change normalized to the baseline I_0_ for a P3HT nanofibers sensor tested versus increasing acetone concentrations (namely, 3.5, 10.5 and 17.6 ppm) in nitrogen as the gas carrier, at 25 °C. The black arrows mark the end of the analyte injection procedure (approximately three seconds prior to observe the sensor response onset; for the 17.6 ppm this time was a bit lower, around 2.5 s). The inset evidences the linear relation between the ΔI_peak_/I_0_ ratio and the considered acetone concentrations for all injections experiments conducted in nitrogen as gas carrier.

**Figure 5:**
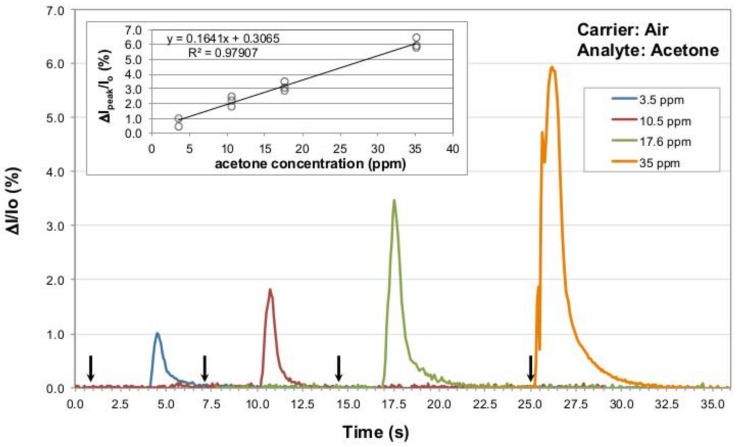
Current percentage change normalized to the baseline current I_0_ for a P3HT nanofibers sensor tested versus increasing acetone concentrations (namely 3.5, 10.5, 17.6 and 35 ppm) in air as the gas carrier, at 25 °C. The black arrows mark the end of the analyte injection procedure (approximately three seconds prior to observe the sensor response onset; for the 17.6 ppm this time was a bit lower, about 2.5 s, and for the 35 ppm the time was extremely short, around 0.1–0.2 s). The inset shows the linear relation between the ΔI_peak_/I_0_ ratio and the corresponding tested acetone concentrations for all injections conducted in air as the gas carrier.

After the preliminary tests, the sensing element has been calibrated using increasing and controlled acetone amounts. In each case the analyte injection time was shorter than the chamber flushing time (the highest amount, 5 mL, required less than three seconds for the injection). Therefore, concentrations of 3.5, 10.5 and 17.6 ppm calculated for the three different acetone injections can be assumed to be actual acetone concentrations reached inside the testing chamber along detection events, and the sensor responses to these concentrations are reported in Figure 4. In order to highlight the sensitivity of the sensor to small acetone concentrations, its response is reported in terms of current percentage change with respect to the baseline I_0_, using the formula ((I_Peak_ − I_0_)/I_0_) × 100, where (I_Peak_ − I_0_) is expressed as ΔI in Figure 4 and Figure 5. Peak intensity-to-baseline ratios derived from signals exhibited by the P3HT NF sensor exposed to repeated gas analyte injections at the considered concentrations are reported in the figure inset. As it can be appreciated, the range of injected gas analyte volumes and flow conditions achieved within the chamber are such that peak intensity-to-baseline ratios are linear with increasing acetone concentrations (R^2^ coefficient of 0.9892 in inset of Figure 4).

As user-friendly sensors for detecting acetone in breath would be normally operated in air, further experiments were done on the same NF devices to assess their response to acetone in air as the carrier gas. Injected gas analyte volumes from 1 up to 10 mL were hence applied in an air stream flowing at 2 L/min. Consequently, acetone concentrations from 3.5 to 35 ppm were estimated to be reached within the testing chamber in this set of tests. The latter concentration value was calculated with an analyte injection time still shorter than the chamber flushing time, but very close to it (5.5 s vs. 5.8 s, respectively), and this point will be discussed in the next section.

The responses of the NF sensor towards these acetone concentrations in air as the gas carrier are shown in Figure 5. Also in this case the signal maxima show a good proportionality to the amount of injected gas (see the linear fit coefficient of 0.9791 in the inset). Moreover, the rise and decay times recorded across these experiments are again in the range of a few seconds.

### 3.4. Assessment of Water Vapor Interference

To assess the possible interference of water vapor in the P3HT NF-based sensing element response, injections with saturated water vapor at 25 °C and ambient pressure using nitrogen as the gas carrier at 2 L/s have been carried out. A clear sensor response to humidity has been appreciated when 5 mL of saturated water vapor were injected in the system (lower amounts of water vapor did not result in a sensor signal easily distinguishable from the baseline noise). At the flow rate used for these tests, 5 mL of saturated vapors of water need about 2.8 s to move through the testing chamber. With this gas travel time the turbulent mixing of the water vapor is ensured, so that 0.61 ppm is a reasonable estimate for the maximum water concentration gained in the testing chamber volume along the experiment.

**Figure 6:**
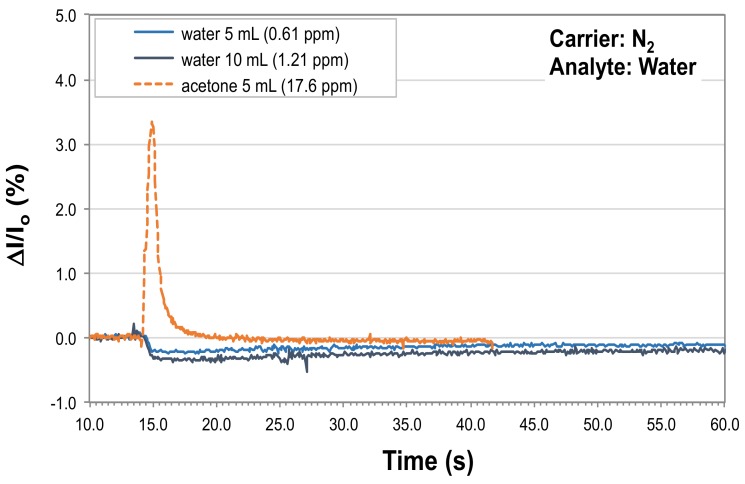
P3HT NF-sensor response to injections of 5 mL (cyan curve) and 10 mL (dark grey curve) of water vapor saturated at 25 °C, using nitrogen as the carrier gas and the same experimental conditions (carrier flow rate, temperature and ambient pressure) used along tests carried out to measure in-flow acetone response of P3HT NF sensing elements. The orange dashed curve represents the response of the sensing element to 5 mL of acetone. In the inset: magnification of the response of the sensing element to 5 and 10 mL of water vapor (cyan and dark grey curve, respectively).

As shown in the inset of Figure 6 (cyan curve), this amount of water vapor produces a response of about −0.22 % ΔI/I_0_ units. This response is opposite in sign, and of a much lower intensity (about one order of magnitude), with respect to that an analogous volume of acetone (see the orange dashed curve of Figure 6, which was overlapped in this graph to highlight the difference in response between the two gases, and was not due to an actual acetone exposure test carried out during the here considered water response assessment). On the other hand, even though the two volumes are equal, they correspond to very dissimilar concentrations, due to the difference in partial pressure between the two gases at 25 °C (about 0.282 atm for acetone, against about 0.0314 atm for water). In addition, the response evidences a long signal tail.

By injecting 10 mL of saturated water vapors, the sensor’s signal change is of about −0.33 % with respect to the baseline, with an even longer tail. This amount of water vapor corresponds to a maximum concentration achieved in the chamber, before the carrier gas flow begins to flush the water away, of 1.21 ppm. The limited difference between this response and the previous one deriving from the 5 mL injection clearly signals that the sensor, in these conditions, is already in saturation: if it would not, doubling the amount of injected water, a two-fold response with respect to the 5 mL injections should have been observed. Therefore, any higher amount of water injected would have resulted in very similar responses.

Since P3HT is hydrophobic, a swelling effect due to water absorption is ruled out as a possible cause of the sensor response to water. The saturation of the P3HT NF sensor upon injection of 10 mL of water vapor is likely due to the fact that the water has a much longer response decay time than the acetone [18,19]. Therefore, by the time that the last injected water vapor is arriving on the sensor (with a 10 mL injection the total injection time is about 5.5 s, close to the 5.8 s needed to completely fill the chamber in the considered flow conditions), the first arrived water vapor is still adsorbed on the P3HT surface, originating signal saturation phenomena. Moreover, 10 mL of analyte corresponds to the upper limit of the chamber linear response, as found with acetone (see Fig. 5 and the related text).

## 4. Discussion

### 4.1. Breath Sensing-Relevant Validation of the Measurement Setup via Flow Injection Analysis

The measurement setup developed in this work was conceived to enable testing sensors in a breath-relevant environment. The two main functional features of breath we considered for the system design and validation step (carried out with the commercial Figaro sensor) are i) the continuous gas flow nature of breath analytes, and ii) the very complex gas mixture composition characterizing actual exhaled breath, which are known to include a large fraction of nitrogen (about 78%), oxygen (about 15%), CO_2_ (about 5%), argon (slightly less than 1%), water vapor (about 10^4^ ppm), and tiny amounts of hundreds of volatile organic compounds [43].

For addressing point (i) we focused on the approach of gas flow injection analysis [31], which uses a gas sensor exposed to a gas stream to detect analyte/analytes mixtures injected in the system, and that has been already explored in the past using metal oxide semiconductor sensors with promising results [32]. The point (ii) was addressed by validating the measurement system with laboratory air as the analyte and nitrogen as the carrier gas. This choice was made because standard environmental air includes a significant amount of water (a relative humidity, RH, of around 60% was measured with a digital hygrometer just before the experiments), oxygen and nitrogen, making it an ideal test analyte for validating a setup aimed at probing sensing elements for breath sensing. Moreover, normal air has the additional advantage of not containing in a meaningful amount the volatile organic compounds (VOCs) usually found in human breath, that could complicate further the analysis of the Figaro sensor’s response.

We hence decided to test the Figaro sensor with nitrogen as carrier gas, which, being an inert gas, offered the further advantage of avoiding any possible interference with the analyte mixture. The analyte air volumes used for the validation tests were chosen in accordance with the intent of characterizing the chamber towards air volumes of interest for breath analysis, i.e., in the mL order of magnitude. Results reported in Figure 3 a, b show that the conceived setup enables detecting the gas analyte passage linearly with respect to increasing injected volumes of the gas analyte. However, it must be pointed out that the Figaro’s signals reach maximum values after tens of seconds, evidencing a rather large time mismatch with respect to the injections times corresponding to 1, 5 and 10 mL lab air volumes (that are 0.5, 2.8 and 5.5 s, respectively, at 1.8 mL/s injection speed), and also with respect to the testing chamber flush time (a bit more than 1 s) at the used carrier flow-rate of 10 L/min. This occurrence has been attributed to a mix of different causes, including the presence of a double stainless steel mesh surrounding the sensor, which could act as a sampling reservoir of the gas mixture stream (hence extending the time length of the analyte/sensing surface interaction), and the chemophysical absorption of the oxygen on the inorganic semiconductor sensor surface, that occurs in largely unoptimized conditions for the Figaro sensor, which is designed to operate in air atmosphere. The double stainless steel mesh would also justify both the time lapse between the analyte injection start and the sensor response onset, that was found to be around three-four seconds, and the elapsed time between the signal peak and its return to the baseline, which is in the order of several hundreds of seconds.

In these conditions, it is not possible to associate reliable analyte concentrations to the signal peaks provided by the Figaro. In any case, as the linearity of the recorded response of the system with respect to the injected volumes of analyte is extremely high (R^2^ = 0.9995, see Figure 3b), the realized setup was considered to be successfully validated and suitable to carry out flow injection analysis tests on gas sensor devices.

### 4.2. Response of the P3HT NF-Based Sensor to Acetone

Even though the whole gas sensing setup was conceived for validating sensors aiming to breath analysis, in this first assessment of the novel P3HT NF-based sensing element we focused on investigating the response of these devices to pure gaseous acetone. This approach was chosen in order to have an evaluation of their performance with respect to a simple single-analyte environment. In this frame, the P3HT NF-based device response to acetone was assessed at first again in nitrogen.

In healthy subjects the acetone concentration in breath is around 0.5 ppm [41], therefore it would have been desirable to go below the ppm limit, a threshold that has not been reproducibly achieved with the current chamber setup. Nonetheless, the lowest acetone concentration limit here tested, i.e., 3.5 ppm, is well in line with values found in diabetes-suffering persons [41], hence it already has clinical relevance. Sensing system setup improvements, like a closed injection loop (that will avoid manual analyte injection procedures) and a more refined data processing approach, that will lower baseline fluctuations, are expected to allow us to reach sub-ppm limit in our system very soon. In any case, already at the 3.5 ppm concentration, the P3HT NF sensor evidenced repeatable performances, with a satisfactory peak-to-baseline noise factor and stable current characteristics over repeated cycles (see Figure 3). This behavior testifies for effective conditioning of the glass syringe and negligible, if any, trapping of acetone as adsorbed molecules onto the syringe’s internal walls, or in other parts of the injection circuit. The observed repeatability is extremely promising for practical applications, especially considering that the acetone injection procedure is fully manual, hence it implies an intrinsic and hardly controllable variability. Moreover, the very low bias at which the sensor is operated (0.5 V) and the low steady current absorption (around 10 nA) lead to very low power consumption, around 5 nW, which further supports the idea of realizing portable devices for personal healthcare applications. Another point of interest for practical applications comes from the observation that the normalized pulse response is characterized by very short rise and fall times, i.e., below 0.5 s and around 2 s respectively, which are compatible with real-time acetone detection in practical, user-friendly breath analyzers, since an average expiration, of about 400-500 mL of exhalate, takes place in 2–3 s [42].

The extremely fast rise time is explained considering that the P3HT nanofibers allow plenty of surface interaction with the incoming gas, making sites for gas/sensing material interaction immediately available to the analyte, with no delay due to progressive adsorption of the gas over the active sensing layer. This effect of nanofibers in gas sensing devices, with respect to their flat or porous counterparts, is frequently reported in literature [7,8,35,36,37,44]. The slightly slower, but still notably fast, fall times are instead attributed to the fact that the ASB-SANS-generated P3HT nanofibers are very compact: in fact, UV-Vis and XRD characterization showed in previous work that the macromolecular chains in these fibers are tightly packed, resulting in a marked crystallinity [39]. In these conditions acetone is not able to penetrate in depth within the core of the nanofibers, leading to the observed very fast signal fall times. The same structural feature of the nanofibers is assumed to be responsible for the high stability of the sensing element in time, despite P3HT is known for being subject to photooxidation. In fact, very compactly organized chains makes it difficult for oxygen molecules to permeate the polymeric chains, making the device overall more robust to degradation. 

Another remarkable feature of ASB-SANS-generated P3HT nanofibers sensors is their very satisfactory linearity (in terms of peak intensity-to-baseline ratio) between 3.5 and 17.6 ppm, as shown in Figure 4. This linearity is verified also when the sensor is tested in air (which is a more realistic analysis environment than nitrogen) as the carrier gas, up to acetone concentrations as high as 35 ppm (Fig. 5), suggesting that even at this relatively high amount of acetone the P3HT NF sensing element actually detects the correct analyte concentration.

The estimated sensitivity of the P3HT NF sensor for acetone in the aforementioned conditions is of 0.192 percentage units/ppm for the device operated in nitrogen, and of 0.164 percentage units/ppm for the sensor operated in air, with the already stated caveat of the flowing gas nature of the whole sensor characterization system, which makes all the concentrations we calculated to be just maximum analyte concentrations during the flow time, rather than steady state ones. The same sensitivities calculated with respect to the injected mL of acetone are 0.57 percentage units/mL and 0.68 percentage units/mL, for the devices operated in nitrogen and in air, respectively.

The tested concentration range has been chosen since acetone concentrations in breath at the onset of a hyperglycemic crisis can be around several units of ppm [44], hence this range has a clinical relevance. Lower acetone concentration values are worth exploring, since in diabetes-suffering people the acetone concentration in breath during normal metabolism (i.e., in absence of hyperglicemic crises) is in the 1–5 ppm range, while in healthy subjects this value is in the tenths of ppm range [41]. On the other hand, at the moment our system, though carefully conceived and able to detect the analyte with reality-compliant rapidity and in truly dynamic, real time conditions, suffers from the manual analyte injection procedure which implies systematic errors and consequent lack of reproducible results below the 3.5 ppm acetone concentration.

### 4.3. Assessment of Water Interference Potential

As seen in Section 3.4, the P3HT NFs-based sensing element can detect water. However, upon the presented data, the influence of water on the response of the P3HT NFs-based sensing element to acetone is believed to be negligible. In fact, the injected acetone samples were prepared in laboratory atmosphere, hence they already contained water from the room humidity (about 60% RH), and this occurrence was found to be irrelevant with respect to the observed sensing element behavior, which is linear, reproducible and reversible. Even at the lowest acetone concentrations of 3.5 ppm (corresponding to an injection of 1 mL) no noticeable trace of long (several tens of seconds, up to two and more minutes, see inset of Figure 6), negative signal decays (signal tails) attributable to water is visible. Only a small negative peak immediately preceding the acetone detection, seen at the lowest acetone concentrations (Figure 3), could be in principle attributed to the small amount of water present in the saturated vapors of acetone (that were prepared in normal laboratory air).

Of course, it could be considered that at 25 °C, the temperature at which the measurements have been carried out, 5 mL of saturated water vapors contain about 6.4 × 10^−6^ moles of water, while at 37 °C, i.e., the temperature of human breath, this quantity is raised by about one order of magnitude, to about 4.3 × 10^−5^ moles, hence in this latter case it is in principle possible that a contribution on the sensing element response due to water would be observed. On the other hand, the P3HT NF sensor is in saturation regime with respect to water already in the tested conditions (saturated water vapor at 25 °C). This means that even by increasing substantially the amount of water arriving on the P3HT NF sensing element the overall observable interference would be very limited.

We attribute this behavior to the known hydrophobicity of P3HT, which in presence of both acetone and water could provide a more favorable energetic landscape for the adsorption of the less polar acetone with respect to the highly polar water. In this picture the readily adsorbed acetone would physically hinder the access to the nanofibers’ surface to the water molecules, leading to the observed apparent absence of meaningful interference. More thorough investigations on this point are in progress.

In terms of practical applications, the evidence that water does not provide meaningful interference to acetone detection would in principle suggest a promising potential for the use of the P3HT NFs-based sensing element as a self-standing sensor for acetone detection in breath. However, due to the non-negligible response of the sensing element to water, it could be more appropriate to consider the P3HT NFs as better suited for constituting a preferential acetone-sensitive element of an electronic nose. In this context, appropriate hardware (i.e., multiple sensing points, each one with its own preferential but not exclusive selectivity) and software (i.e., algorithms capable of processing the data from the different sensing points constituting the array) can allow good breath sensing properties even in the absence of strict selectivity to acetone of the P3HT NFs sensing element.

### 4.4. Investigations Over the Sensor Working Mechanism

As stated in Section 4.2, the general sensing mechanism of the P3HT NF is believed to be based on the adsorption of the analyte onto the nanofibers’ surface [7,8,35,36,37,44]. A deeper insight into the detection mechanism can be made considering that the baseline recovery times of P3HT NF sensors have similar decays, independently from using nitrogen or air as carrier gases (see Figure 4 and Figure 5).

**Figure 7:**
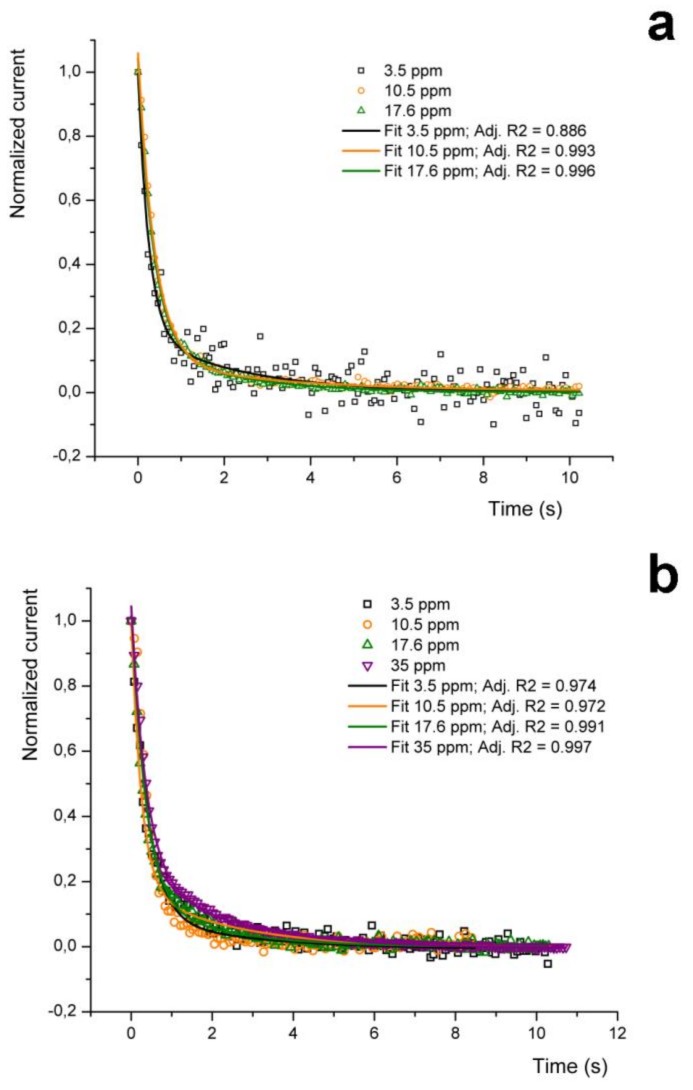
(**a**) Normalised current plots representing descending P3HT nanofibers sensor’s traces after exposure to the gas analyte (acetone at 3.5, 10.5 and 17.6 ppm) in nitrogen as the carrier gas, at 25 °C. (**b**) Normalised current plots representing descending portions of P3HT nanofibers sensor’s traces after exposure to the gas analyte (acetone at 3.5, 10.5, 17.6 and 35 ppm) in air as the carrier gas, at 25 °C.

In particular, as shown in Figure 7, the sensor pulse response is characterized by a fall time from 90 down to 10% of the peak value (Baseline Recovery Time, BLRT) of about 1 s at all considered concentrations and under any type of carrier gas, except for the 35 ppm concentration under air as carrier, which is around 2 s.

This latter difference is explained by considering that at these concentrations the P3HT NF-based sensing element seems to reveal the mismatch between the testing chamber flushing time and the injection time associated to this acetone amount (which is about 5.5 s), as suggested also by the fragmented rise signal trace, by the slightly increased pulse rise time of the corresponding peak and by the extremely short time elapsed between the end of the analyte injection and the sensor response onset (Figure 5). These conditions can be hence considered as the onset of a non-linear sensor behavior due to excessive analyte amount injected in the chamber. On the other hand, the overall device response at this concentration is still satisfactorily linear (Figure 5, inset), which suggests that 35 ppm might be an upper acetone concentration limit for this setup.

The observed relative uniformity in the BLRTs of the responses to different amounts of acetone between different carrier gases was unexpected. To better verify this behavior, the decay of the NF sensor signals collected using nitrogen as the carrier gas have been fitted with a bi-exponential equation of the type *y* = *y_0_* + A_1_ exp(−*x*/t_1_) + A_2_ exp (−*x*/t_2_) (Figure 7a, black, orange and green continuous plots, corresponding respectively to 3.5, 10.5 and 17.6 ppm). As reported in Table 2, the adjusted R^2^ values are rather satisfactory in almost all the cases, and just the 3.5 ppm in nitrogen data set evidences a relatively lower value (0.886, against an average higher than 0.9), due to the noisier signal at this low concentration (Table 2). The same fit equations have been hence applied to the corresponding acetone concentrations data set obtained using air as carrier gas (leaving just the fit curve offset, y_0_, as a floating parameter to allow convergence; Figure 7b). This procedure delivered a surprisingly good fitting also in these cases (for each concentration the adj. R^2^ was > 0.97), despite the different carrier gas used for the detection.

This intriguing finding strongly suggests that the desorption phenomenon occurring under nitrogen is very similar to that occurring under air, pointing to a predominant role of the acetone-P3HT interaction in shaping the decay time of the NF sensor signal, rather than to a significant effect of the surrounding atmosphere. This view is supported by previous findings that showed a correlation between the 90% decay time of signals generated by different analytes and their respective polarizabilities [18]. We assume that this phenomenon is due to the concurrent poor solvent capability of acetone with respect to P3HT [45] and high crystallinity of the ASB-SANS-produced P3HT NFs [39], both factors that impede a meaningful penetration of acetone within the nanofibers. In fact, the poor solvent capability of acetone for P3HT implies its accumulation at the nanofibers’ surface, rather than an interaction with the inner part of the nanofibers, leading hence to a fast stripping of the solvent from the nanofibrs’ surface by the continuously flowing carrier gas, independently from the chemical nature of the latter. In addition, highly crystalline P3HT hinders the penetration of acetone within the fibers, minimizing the analyte interactions with the polymer and further contributing to a fast desorption.

This peculiar atmosphere-independent behavior suggests that highly crystalline nanofibers produced out of different polymers can be used as sensing elements in electronic nose-like devices aimed to real time breath analysis, delivering fast responsive, low-cost and highly portable devices. Indeed, ASB-SANS was already demonstrated able to produce such nanofibers out of several different polymers, like poly(methylmethacrylate), PMMA [38,40], poly(L-lactic acid), PLLA [46], and other yet unpublished materials like poly(lactic-co-glycolic acid, PLGA) or molecular compounds, like PCBM (a fullerene derivative), providing to such devices a notable versatility and ready industrializability.

Furthermore, the good fitting obtained by using a bi-exponential decay could reflect a desorption mechanism due to two different physical phenomena: a first, fast desorption of a multilayer of acetone molecules weakly adsorbed on the nanostructures, and a second slower desorption of an acetone monolayer more tightly adsorbed on the nanofibers’ surface. This hypothesis is supported by several findings over this type of behavior of water and other polar molecules (formaldehyde, methanol, ethanol, etc) in simulations of gas desorption from interstellar grains, as measured by Thermal Programmed Desorption (TPD) [47,48], and will be further investigated in future work.

## 5. Conclusions

A testing setup based on flow injection analysis has been realized and validated using a commercial sensor in dynamic gaseous flow, breath analysis-relevant conditions. P3HT nanofibers-based sensors have been fabricated and tested towards acetone in the validated system. Tests were hence carried out with acetone as the injected analyte, using either dry nitrogen or dry air as the carrier gas. The devices demonstrated rather reproducible results with respect to amounts of acetone as low as 3.5 ppm, and linear responses along analyte concentrations from 3.5 to 17 ppm (in nitrogen) and from 3.5 to 35 ppm (in air). Other general features of P3HT NFs sensors include excellent baseline recovery capabilities (i.e., remarkable reversibility), even upon repeated exposures to the analyte, no observable current drift upon analyte detection, and very short pulse rise and fall times (less than 1 s and about 2 s, respectively). The devices were found to have a minimal response to significant amounts of saturated water vapors. This response, though detectable, was not found to be able to affect in an appreciable way the overall response of the sensing elements to acetone.

From a more fundamental point of view, the time decay dynamics of the P3HT NFs signals were found to be extremely similar for operation under nitrogen and air, which can be described by a bi-exponential decay law. This behavior can be ascribed to a first rapid elimination of the external multilayer of gaseous analyte molecules, followed by a relatively slower step of progressive desorption of the acetone monolayer directly adhering onto the P3HT nanofibers surface, all aided by the continuous carrier gas flow, which likely contribute to strip the analyte away from the nanofibers. Previous findings on the same type of sensors pointing to a relation between this parameter and the analyte polarizability suggest a detection mechanism operating via polarization effects of the surface *ad opera* of the analyte. The observed apparent independency of the decay times from the type of used carrier gas indicates that the P3HT/analyte interactions greatly overcome those between P3HT and the gaseous carrier, further supporting the observations over the negligible interference of water in the detection of acetone.

Overall, the described features of the P3HT NFs sensing element, coupled to its very low power consumption (around units of nW) and to its small physical footprint (about 1 cm^2^), are very promising for its practical application in acetone sensing for breath analysis. However, as the P3HT NFs has some limited sensitivity also to water, the suggested application is that of a sensing element of an electronic nose, rather than a self-standing sensor. As the selectivity of the nanofibers-based sensing element can be tuned via a variety of readily available chemical (surface functionalization, changes in the polymer type) and physical (tuning of the nanofibers morphological characteristics, like size or density) techniques, the here proposed approach for realizing nanostructured sensing elements for highly portable, real time, low-cost, lightweight analyzers for breath’s volatile organic compounds holds promise for a wide applicability of the here described device.

## Figures and Tables

**Table 1:** Summary of the different conditions used for the described experiments.

Type of Sensor (Figaro = Validation; P3HT NFs = Measurement)	Type of Carrier Gas	Carrier Gas Flow Rate	Analyte	Tested Amounts of Analyte
Figaro	Dry nitrogen	10 L/min	Laboratory air (approx. water content: 0.61 ppm)	1, 5, 10 mL *
P3HT NFs	Dry nitrogen	2 L/min	Saturated acetone vapors	3.5, 10.5, 17.6 ppm
P3HT NFs	Dry air	2 L/min	Saturated acetone vapors	3.5, 10.5, 17.6, 35 ppm
P3HT NFs	Dry nitrogen	2 L/min	Saturated water vapors	0.61, 1.2 ppm

* for the validation measurement the amount of injected analyte is given in mL, rather than in ppm, because of the very long times of desorption of the analyte from the sensor’s surface, which made any attempt to calculate the actual concentration within the chamber not significant.

**Table 2:** Fitting curve parameters for the tested acetone concentrations under different carrier gases.

		Acetone Concentration (ppm)						
		3.5		10.5		17.6		35
	Carrier Gas	Nitrogen	Air	Nitrogen	Air	Nitrogen	Air	Air
Fit Parameter								
y_0_		−2.517 × 10^−4^	−0.00262	0.00625	−0.00856	−0.00507	−0.00109	−0.00447
A_1_		0.808	0.808	0.950	0.950	0.925	0.925	0.246
t_1_		0.247	0.247	0.384	0.384	0.394	0.394	2.26
A_2_		0.189	0.189	0.103	0.103	0.102	0.102	0.802
t_2_		2.29	2.29	2.72	2.72	3.11	3.11	0.355
Adj. R^2^		0.886	0.974	0.993	0.972	0.995	0.988	0.997
